# Adoption of a gastroenterology hospitalist model and the impact on inpatient endoscopic practice volume: a controlled interrupted time-series analysis

**DOI:** 10.1016/j.igie.2024.04.008

**Published:** 2024-04-25

**Authors:** Dennis Shung, Darrick K. Li, Kisung You, Kenneth W. Hung, Loren Laine, Michelle L. Hughes

**Affiliations:** 1 Section of Digestive Diseases, Department of Internal Medicine, Yale School of Medicine, New Haven, Connecticut, USA; 2 Department of Internal Medicine, Yale School of Medicine, New Haven, Connecticut, USA; 3 VA Connecticut Healthcare System, West Haven, Connecticut, USA

## Abstract

**Background and Aims:**

The gastroenterology (GE) hospitalist staffing model has multiple potential benefits for the inpatient and outpatient care of GE patients. The GE hospitalist model may improve inpatient endoscopy efficiency via better provider familiarity with management of GE emergencies, hospital systems, and workflow, and may also increase outpatient endoscopy capacity by decreasing the need for inpatient coverage by outpatient providers. However, the real-world impact of this model on inpatient and outpatient endoscopic volume remains uncertain.

**Methods:**

We conducted a controlled interrupted time-series analysis from September 2018 to March 2020 comparing inpatient endoscopy volume at 2 high-acuity hospitals within the same academic health system, one of which adopted a 2-physician GE hospitalist model in July 2019. We also performed a single interrupted time-series analysis of outpatient endoscopic volume of the practice employing GE hospitalists.

**Results:**

After implementation of the GE hospitalist model, weekly volume of inpatient endoscopic procedures increased by 10.9 (95% CI, .6-21.2; *P* = .024) compared with a hospital using traditional staffing. Outpatient endoscopic procedure volume also increased by 39.8 per week (95% CI, −5.78 to 85.44; *P* = .09), with no change in the number of physicians performing endoscopy.

**Conclusions:**

Our findings demonstrate that introduction of a GE hospitalist model increased inpatient and outpatient endoscopic volume in a large academic center.

Hospitalists have become common and are associated with improved quality, decreased costs, and increased productivity.^1^, ^2^, ^3^, ^4^ Gastroenterology (GE) hospitalists have only recently, however, started to gain popularity.^5^ Although a diversity of models exist, usually 1 or 2 GE hospitalists manage inpatient GE consultation services and perform associated endoscopies.^6^ Despite growing interest, there is a dearth of literature examining the impact of these models.^7^^,^^8^ Conceptually, it is thought that GE hospitalists would increase a practice’s productivity.^9^ However, no real-world data yet exist, and quantifying the impact remains essential. In the present study, we provide the first data illustrating the impact of a GE hospitalist model on endoscopic volume in an academic practice.

## Methods

### Study design

We conducted a retrospective observational cohort study of patients from September 2018 to March 2020 at 2 urban academic hospitals within the Yale–New Haven Health (YNHH) system that share similar patient populations: Yale–New Haven Hospital, York Street Campus (YSC), a 1021-bed hospital, and Bridgeport Hospital (BH), a 501-bed hospital. A controlled interrupted time-series analysis was performed to determine the impact of a GE hospitalist model on inpatient endoscopy volume (primary outcome). For the primary outcome, BH served as control. A single interrupted time-series analysis was also performed evaluating the impact of a GE hospitalist model on outpatient endoscopic volume of the YSC academic practice. An interrupted time series is considered to be a strong quasiexperimental design to assess whether a difference in outcomes occurred after intervention compared with the preintervention period serving as the control. Information regarding all inpatient and outpatient endoscopic procedures performed at each site was obtained from Provation (Minneapolis, Minn, USA). Data collection was stopped on March 1, 2020, to avoid a potential confounding impact from the Covid-19 period.

### Intervention

The GE hospitalist model began in July 2019 at YSC when 2 GE hospitalists were employed full-time to cover inpatient general GE consult service. Both rotated responsibilities weekly, one completing inpatient procedures from 8 am to 12 noon, and one covering consultations from 8 am to 5 pm plus afternoon procedures. Before this intervention, the service was staffed by a faculty member at 1-week intervals. During time on service, this provider’s outpatient clinic and endoscopy sessions were suspended so that they could staff consultations and follow-ups and perform procedures throughout the day as needed. After implementation, the service was covered by GE hospitalists, with other GE faculty rotating only 2 weeks per year. After-hours coverage remained unchanged. At BH, the inpatient service was provided by a combination of 4 attending physicians covering for their respective practices at any given time throughout the intervention period.

### Statistical analysis

Segmented linear regression with autoregressive error models was performed to account for trends at baseline, before and after implementation of GE hospitalists as previously described.^10^ Additional details are provided in Appendix 1 (available online at www.igiejournal.org). Models were tested for autocorrelation by means of the Cumby-Huizinga test, and detected autocorrelation was addressed with the use of Newey-West standard errors. Results are reported with 95% confidence intervals (CIs) and *P* values. As a sensitivity analysis, we performed another controlled interrupted time-series analysis using data from Yale–New Haven Hospital, St Raphael’s Campus (SRC) as control, a 520-bed hospital within the YNHH system with a similar patient population that did not adopt a GE hospitalist model. Coverage for inpatient service both before and after the intervention was provided by 2 affiliate gastroenterologists who provided coverage for their respective practice’s patients. Providers would suspend outpatient endoscopy and clinic sessions while covering the SRC inpatient service. All analyses were performed with the use of Stata v17.0 (StataCorp, College Station, Tex, USA).

## Results

### Inpatient endoscopy volume

**Figure 1:**
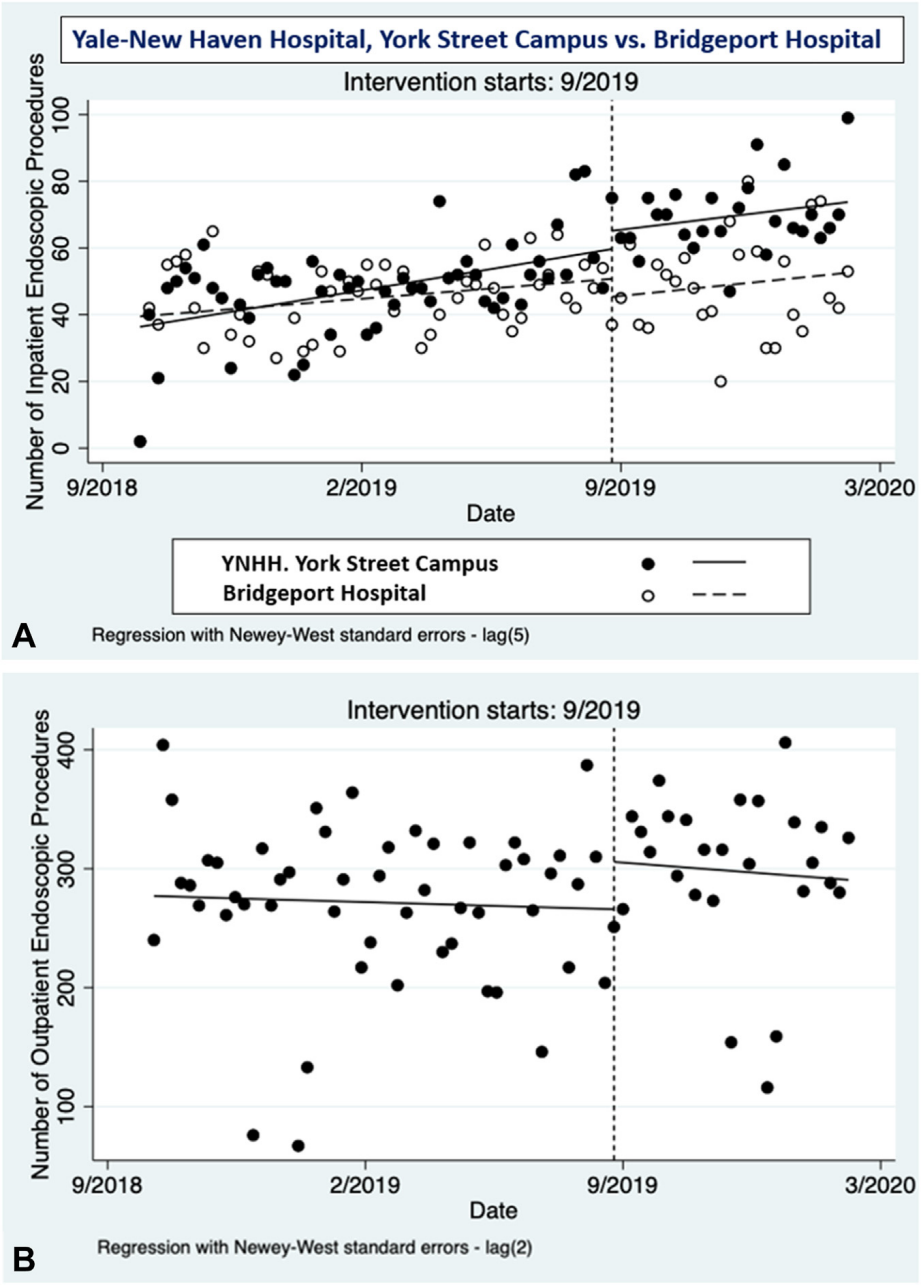
Interrupted time series analyses of inpatient and outpatient endoscopic volume. **A,** Controlled interrupted time-series analysis comparing inpatient endoscopic volume between Yale–New Haven Hospital, York Street Campus and Bridgeport Hospital with implementation of GE hospitalists at the former. **B,** Single interrupted time-series analysis of outpatient endoscopic volume of the Yale Section of Digestive Diseases.

**Supplementary Figure 1:**
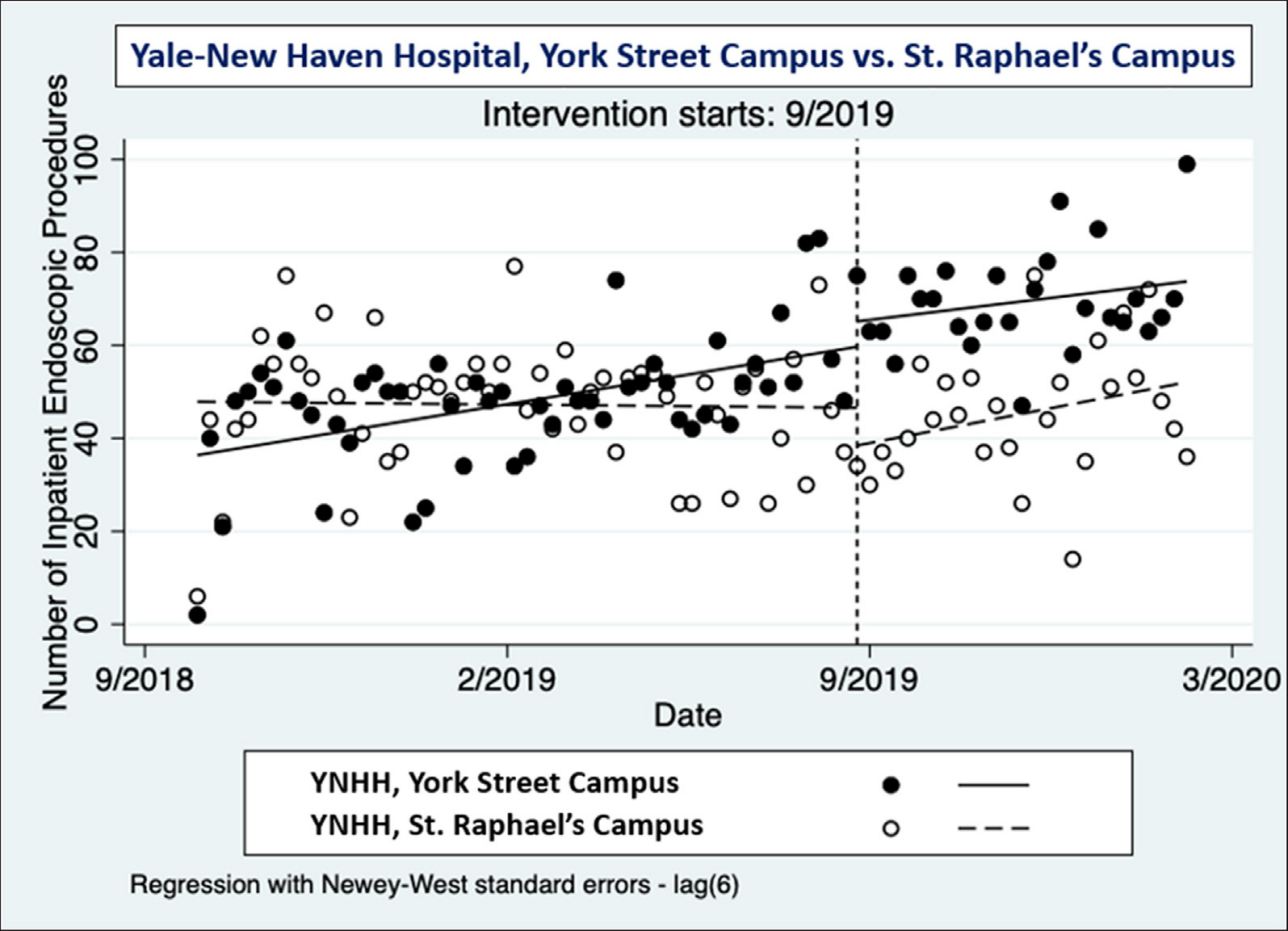
Controlled interrupted time series of inpatient endoscopic volume comparing Yale–New Haven Hospital’s York Street Campus and St Raphael’s Campus.

**Supplementary Figure 2:**
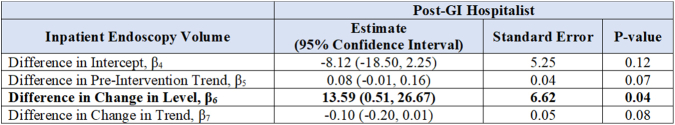
Sensitivity analysis of controlled interrupted time series analysis using different control group. Intervention group was Yale–New Haven Hospital, York Street Campus. Control group was Yale–New Haven Hospital, St Raphael’s Campus. Difference in level or trend is the relative to control group.

Controlled interrupted time series evaluating inpatient endoscopy volume at YSC and BH are presented in Figure 1A. Before introduction of GE hospitalists, baseline weekly inpatient endoscopic volume trend (0.01; 95% CI, −0.05 to 0.07; *P* = .76) and level (−5.42; 95% CI, −11.50 to 0.65; *P* = .08) were similar between YSC and BH. After introduction, there was an increase of 10.9 endoscopic procedures per week compared with BH (95% CI, 0.6-21.2; *P* = .04) (Table 1). Difference in change in pre-/postintervention trends was −0.03 (95% CI, −0.12 to 0.07; *P* = .58). Sensitivity analysis comparing YSC and SRC was similar, with an increase in at YSC compared with SRC of 13.6 endoscopic procedures per week (95% CI, 0.5-26.7; *P* = .04) after implementation (Supplementary Figs. 1 and 2, available online at www.igiejournal.org).

**Table 1:** Association of GE hospitalist model with inpatient and outpatient endoscopic productivity

	After GE hospitalist		
	Estimate (95% CI)	SE	*P* value
Inpatient endoscopy volume			
Difference in intercept, β*4*	-5.42 (-11.50 to .65)	3.07	.08
Difference in preintervention trend, β_5_	0.01 (-.05 to .07)	.03	.76
Difference in change in level, β_6_	10.89 (.58 to 21.20)	5.22	.04
Difference in change in trend, β_7_	-0.03 (-.12 to .07)	.05	.58
Outpatient endoscopy volume			
Intercept, β_0_	277.08 (235.43 to 318.73)	20.90	<.01
Preintervention trend, β_1_	-0.03 (-.21 to .15)	.09	.73
Change in level, β_2_	39.83 (-5.78 to 85.44)	22.39	.09
Change in trend to β_3_	-0.05 (-.43 to .33)	.19	.74

For inpatient endoscopy volume (controlled interrupted time series analysis), difference in level or trend is relative to the control group. For outpatient endoscopy volume (single interrupted time series analysis), difference in level or trend is relative to the preintervention values.

*CI*, Confidence interval; *SE*, standard error.

### Outpatient endoscopy volume

Single interrupted time series evaluating outpatient endoscopy volume for YSC academic practice are presented in Figure 1B. Total numbers of GE physicians performing outpatient endoscopy were equal before and after introduction of GE hospitalists (n = 29; the 2 GE hospitalists were excluded from this count, because they did not perform outpatient endoscopies or have outpatient endoscopy sessions). After introduction of GE hospitalists, there was an increase in of 39.8 endoscopic procedures per week (95% CI, −5.77 to 85.44; *P* = .09) (Table 1). Difference in change in pre-/postintervention trend was −0.05 (95% CI, −0.43 to 0.33; *P* = .74).

## Discussion

Using interrupted time-series analysis, we provide comprehensive data showing adoption of a GE hospitalist model was associated with increases in inpatient and outpatient endoscopic volumes. Our findings add to existing evidence for improved practice productivity associated with use of dedicated inpatient providers.^2^^,^^4^

At our center, adopting GE hospitalists led to significant increase in number of inpatient procedures performed. This is attributed to several factors. GE hospitalists are inpatient based, allowing them to be consistently on site throughout the day and readily available for add-on or emergency cases requiring same-day endoscopy. Moreover, inpatient-focused providers are likely to have more experience with complex, acutely ill patients and more comfort performing challenging cases. Although the added GE hospitalists did not complete additional advanced endoscopy training, they had experience or sought additional training for inpatient-focused endoscopic procedures, such as enteral access and advanced hemostasis techniques, to allow them to do a range of procedures other non-GE hospitalist providers may be less comfortable doing while rotating on service. Increased inpatient endoscopic throughput is also thought to contribute due to improved workflows, closer trainee supervision, and improved communication between GE consultants and other services fostered by a consistent GE hospitalist presence.

Our data show that adopting GE hospitalists numerically increased outpatient endoscopic volume. In most U.S. practices, gastroenterologists primarily work in outpatient settings. However, depending on practice structure, providers must suspend their outpatient practice while on inpatient service or juggle inpatient coverage with clinics and outpatient procedures. This can lead to significant frustration, as reflected by a recent survey that found 81% of GE departments that have added GE hospitalists or were thinking about adding them did so to reduce burnout and time on service for other GE faculty members.^11^ Introduction of GE hospitalists allowed outpatient providers to minimize disruption to their routine practice, thereby improving patient access, optimizing use of endoscopy sessions, and allowing increased procedural volume. In our model, there were relatively few weeks when outpatient providers completely suspended outpatient sessions to cover inpatient service (8%) or decreased outpatient duties to cover morning-only sessions (38%) once GE hospitalists were employed.

Limitations to our study bear discussion. Given the study design, we were unable to control for every variable affecting endoscopy volume, and therefore selection and confounding biases may influence our findings. However, during the time of intervention, there was no substantial change in the number of faculty, inpatient team composition, hospital facilities, or insurance contracts. Moreover, the remaining possible time-varying confounders (eg, population demographics and patient acuity) are likely to be relatively slow to change compared with the duration of intervention and follow-up. Furthermore, any change in demographics and patient acuity, particularly for YSC and SRC, which serve similar populations and are in close geographic proximity, can be reasonably expected to affect both equally. Although there was a smaller than expected difference in baseline inpatient procedural data before the intervention, it was attributed to the higher capacity for both procedural and consultation volume created by the coverage structure at BH, which had 4 attending physicians covering at any given time compared with 1 at YSC, rather than the presence of unaccounted-for variables.

This study is also limited by a short postintervention assessment period of 6 months, which was necessary owing to confounding impacts of the Covid-19 period, and as such we could not determine if the impacts are durable over time. Finally, this study evaluated a single model at a large tertiary academic hospital. Future studies are needed to establish the reproducibility of our model in other hospital settings and to explore the relative impact of different GE hospitalist models.

In conclusion, this study shows that GE hospitalists can be a beneficial staffing strategy in a large academic practice to improve inpatient endoscopic volume, a limited resource which can become a barrier to quality care for hospitalized patients. Further research is needed to examine the effects of GE hospitalists over time and in varied settings.

## Disclosure

All authors disclosed no financial relationships.

## Appendix 1. Model for interrupted timed series analysis

For a single interrupted timed series analysis, the regression model assumes the following form:

$$Y=\beta_{0}+\beta_{1}T_{t}+\beta_{2}X_{t}+\beta_{3}{XT}_{t}$$

*Y* is the outcome (endoscopic procedures per week). *T*_*t*_ is the time since the start of the study, *X*_*t*_ is an indicator variable representing the intervention, and *XT*_*t*_ is the interaction between time and intervention. *β*_0-3_ are coefficients: *β*_0_ is the intercept or value of the outcome at the start of the study period, *β*_1_ is the change in outcome per unit time (trend) before the intervention, *β*_2_ is the immediate step change in level following the intervention, and *β*_3_ is the change in trend following the intervention (relative to the trend before the intervention − *β*_1_). *β*_1_ and *β*_3_ can therefore be summed to provide the trend following the intervention.

For a controlled interrupted timed series analysis, the regression model assumes the following form:

$$Y=\beta_{0}+\beta_{1}T_{t}+\beta_{2}X_{t}+\beta_{3}{XT}_{t}+\beta_{4}Z+\beta_{5}{ZT}_{t}+\beta_{6}{ZX}_{t}+\beta_{7}Z{XT}_{t}$$

*Z* is a dummy variable that delineates the cohort assignment (control or treatment), and *ZT*_t_, *ZX*_t_, and *ZXT*_t_ are additional interaction terms. *β*_0-3_ are coefficients of the control cohort (eg, Bridgeport Hospital or Yale–New Haven Hospital, St Raphael’s Campus): *β*_0_ is the intercept or value of the outcome at the start of the study period, *β*_1_ is the change in outcome per unit time (trend) before the intervention, *β*_2_ is the immediate step change in level following the intervention, and *β*_3_ is the change in trend following the intervention (relative to the trend before the intervention − *β*_1_). *β*_1_ and *β*_3_ can therefore be summed to provide the trend following the intervention. *β*_4-7_ are coefficients representing the difference between the case series (eg, Yale–New Haven Hospital, York Street Campus) and the control series: *β*_4_ is the difference in intercept level, *β*_5_ is the difference in trend before the intervention, *β*_6_ is the difference in immediate change in level following the intervention, and *β*_7_ is the difference in change in trend following the intervention. *β*_5_ and *β*_7_ can be summed to provide the difference in trend following the intervention.
